# Fully integrated, fully automated generation of short tandem repeat profiles

**DOI:** 10.1186/2041-2223-4-16

**Published:** 2013-08-06

**Authors:** Eugene Tan, Rosemary S Turingan, Catherine Hogan, Sameer Vasantgadkar, Luke Palombo, James W Schumm, Richard F Selden

**Affiliations:** 1 NetBio, 830 Winter Street, Waltham, MA 02451, USA

**Keywords:** Automation, CODIS, Microfluidics, Polymerase chain reaction, Rapid DNA analysis, Short tandem repeat

## Abstract

**Background:**

The generation of short tandem repeat profiles, also referred to as ‘DNA typing,’ is not currently performed outside the laboratory because the process requires highly skilled technical operators and a controlled laboratory environment and infrastructure with several specialized instruments. The goal of this work was to develop a fully integrated system for the automated generation of short tandem repeat profiles from buccal swab samples, to improve forensic laboratory process flow as well as to enable short tandem repeat profile generation to be performed in police stations and in field-forward military, intelligence, and homeland security settings.

**Results:**

An integrated system was developed consisting of an injection-molded microfluidic BioChipSet cassette, a ruggedized instrument, and expert system software. For each of five buccal swabs, the system purifies DNA using guanidinium-based lysis and silica binding, amplifies 15 short tandem repeat loci and the amelogenin locus, electrophoretically separates the resulting amplicons, and generates a profile. No operator processing of the samples is required, and the time from swab insertion to profile generation is 84 minutes. All required reagents are contained within the BioChipSet cassette; these consist of a lyophilized polymerase chain reaction mix and liquids for purification and electrophoretic separation.

Profiles obtained from fully automated runs demonstrate that the integrated system generates concordant short tandem repeat profiles. The system exhibits single-base resolution from 100 to greater than 500 bases, with inter-run precision with a standard deviation of ±0.05 - 0.10 bases for most alleles. The reagents are stable for at least 6 months at 22°C, and the instrument has been designed and tested to Military Standard 810F for shock and vibration ruggedization. A nontechnical user can operate the system within or outside the laboratory.

**Conclusions:**

The integrated system represents the first generation of a turnkey approach to short tandem repeat profiling and has the potential for use in both the field (for example, police booking stations, the battlefield, borders and ports) and the forensic laboratory.

## Background

A powerful and reliable tool available today in human identification is short tandem repeat (STR) typing [1-9]. The well-established utility of STR analysis has inspired a desire to accelerate and simplify profile generation for current and novel applications of the technology. For example, the technology would be useful in police stations to determine, prior to suspect release, whether an individual may be associated with crime scene evidence. In immigration offices, it could be applied to support or reject claims of familial relatedness used to justify permission to immigrate, while at borders and ports, it would help to determine whether individuals detained while entering the country illegally have profiles in terrorist DNA databases. Moreover, it would find many applications in military settings, such as to distinguish friend from foe in combat, to permit access through military checkpoints, and to determine attribution of enemy munitions and weapons. Such applications are not currently performed because the processing of DNA samples requires highly skilled technical operators (trained in sample preparation, molecular biology, and data analysis) and a controlled laboratory environment and infrastructure with several specialized instruments. Furthermore, the time between sample collection in the field and obtaining a result in the laboratory is currently too long to allow real-time decisions and dispositions in police, immigration, border, and military applications.

The development of a field-deployable, rapid, fully integrated system for the automated generation of DNA fingerprints has the potential to address both increased demand and expanding applications. The Department of Defense, the Federal Bureau of Investigation, and the Department of Homeland Security developed a series of requirements for such a rapid DNA analysis system, termed ‘ANDE’ (accelerated nuclear DNA equipment [10]). These requirements led us to develop a fully integrated rapid DNA analysis system with the following properties:

1. Ease of use for nontechnical operators: to allow DNA analysis to be performed by a nontechnical operator outside of the laboratory (thereby reducing time to obtain and take action on the result), the system should not require the operator to perform any manual processing steps, reagent loading, assembly, or maintenance.

2. Match or no-match reporting: the information critical to the operator should be provided in a straightforward fashion to allow prompt decision-making (for example, the STR profile generated from an individual is reported as a match or no-match against a given database).

3. Rapid time to result: to have a practical impact on individual processing in field-forward settings, such as a police-station booking desk, the result should be available within 90 minutes.

4. Minimal space and environmental requirements: all processes should be performed in a single instrument, avoiding the need for centrifuges, thermal cyclers, and electrophoresis instruments, and the system should not require a controlled laboratory environment or separated pre- and post-PCR environments.

5. Ruggedization: the system must withstand transport for certain applications (or movement from one part of a room to another) without recalibration.

6. Unitary consumable: to minimize operator time, training, and potential for error, a single cartridge containing all necessary materials and reagents should be utilized. The cartridge should be closed and disposable to minimize sample contamination and user exposure.

7. Data and sample security: as the results of STR analysis can have a profound impact on the individuals being tested, it is critical that privacy rights are respected.

8. Platform technology: many sample types and assays will be required as out-of-laboratory uses of rapid DNA analysis expand; accordingly, a platform technology with modular elements should form the basis of the system.

9. Performance: most importantly, DNA profile quality must meet conventional performance standards for features including concordance, resolution, precision, and peak height balance.

Several groups are working toward fully integrated systems for STR profile generation. Bienvenue *et al*. [11] reported on the partial integration of the process, incorporating DNA purification and polymerase chain reaction (PCR) amplification on a microfluidic device. Their chip is fabricated from glass, requires complex manual manipulations (for example, placing mineral oil over microfluidic PCR chambers), and does not incorporate reagent handling; these are all challenges for developing a field-forward system. El-Sissi *et al*. [12] developed a system that accepts buccal swabs and performs STR analysis. The system requires the insertion of approximately five cartridges for one run, requires refrigerated reagent storage, and performs electrophoresis in glass capillaries, similarly limiting field-forward application. The most advanced system, described by Hopwood *et al*. [13], is based on a disposable plastic cartridge that incorporates DNA purification and amplification coupled to a glass capillary electrophoresis chip for fragment separation. This system requires off-instrument pre-processing of the swab to generate lysate for insertion into the system and requires manual reagent loading.

Here we report a fully integrated, ruggedized, STR analysis system capable of field-forward operation by a nontechnical operator following minimal training. The system employs a consumable-containing, single disposable microfluidic biochip and a fully integrated instrument to perform STR analysis with four fluorescent dyes. The assay interrogates 15 STR loci and the amelogenin gender identification locus. Following insertion of between one and five buccal samples into a BioChipSet cassette (BCSC) and of the BCSC into the instrument, the system performs all required processes for STR analysis for each sample including DNA purification, PCR amplification, electrophoretic separation, fluorescence detection, and data analysis by the on-board expert system to generate a profile. The resulting information is available in one of three electronic files that permit direct viewing of the DNA profile, its re-analysis in traditional STR analysis software, and submission of the results directly to relevant databases.

## Methods

### BioChipSet cassette design

**Figure 1:**
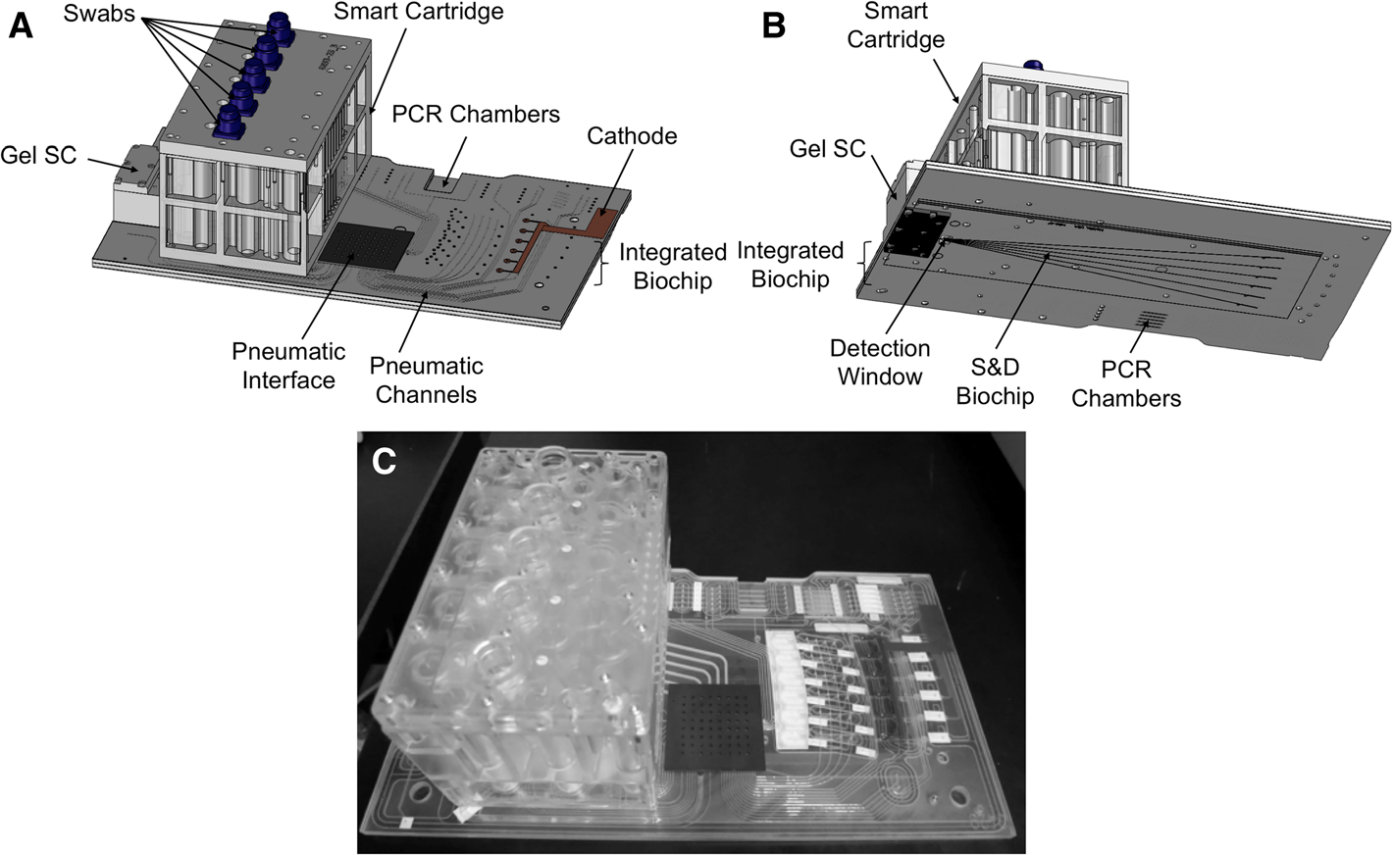
**BioChipSet cassette. (A)** Top view schematic showing the location of the smart cartridge, gel smart cartridge, and integrated biochip. **(B)** Bottom view schematic showing the S&D biochip. **(C)** Photograph. Primary interfaces with the instrument are via the pneumatic manifold, PCR chambers, cathode and anode (not shown, adjacent to the gel smart cartridge), and S&D window. S&D, separation and detection; SC, smart cartridge.

The BCSC is injection-molded using cyclic olefin polymer and is a single-use, disposable device with all reagents factory preloaded. It has four major components (Figure 1):

•The smart cartridge is the largest component, and is a block of 93 × 152 × 84 mm. The smart cartridge consists of five separate purification units, each unit accepting a buccal swab. The custom swab has a DNA-free cotton head (The Bode Technology Group, Lorton, VA), a reinforced plastic shaft, and a cap that contains a radio-frequency identification (RFID) chip for sample tracking. The swab irreversibly locks into the swab chamber. The smart cartridge contains a single formamide storage reservoir, and each of the five units of the smart cartridge contains four reservoirs to hold liquid purification reagents, giving a total of 21 reagent storage reservoirs per smart cartridge.

•The gel smart cartridge (33 × 56 × 28 mm) contains the sieving matrix and electrophoresis buffer used for microfluidic separation and detection (S&D). The linear polyacrylamide-based matrix is stored in the gel smart cartridge until required and then loaded just prior to pre-electrophoresis.

•The integrated biochip (166 × 296 × 5.5 mm) consists of two plates and contains microfluidic channels and chambers that represent the heart of the BCSC. It works with the smart cartridge to perform purification by providing means for transfer of liquids from chamber to chamber of the smart cartridge. At the conclusion of the purification process, the integrated biochip accepts purified DNA from the smart cartridge. The integrated biochip contains the lyophilized reagents that are reconstituted during processing to perform PCR and provides electrophoresis-ready samples to the S&D biochip for electrophoresis.

•The S&D biochip (254 × 84 × 0.376 mm) performs size separation of STR fragments by electrophoresis. It receives sieving matrix from the gel smart cartridge.

The BCSC has several critical interfaces with the fully integrated instrument. The pneumatic interface is located at the pneumatic manifold, where pneumatic drive lines from the instrument are clamped to a set of pneumatic and fluidic channels in the BCSC. There is a thermal interface between the PCR region of the BCSC and the thermal cycler, and a second thermal interface between the S&D biochip and the S&D heater. There is a high-voltage interface at the anode and cathode, enabling electrophoresis. Finally, the optical interface is located at the S&D detection window, in which six electrophoresis channels receive laser light to excite fluorescent dyes for detection.

### Reagent storage and release system

The BCSC comes preloaded with all reagents; the user loads neither the instrument nor the BCSC with reagents. Within the smart cartridge and gel smart cartridge, liquid reagents are stored in reservoirs and are contained by aluminum foils bonded to both ends. When the reagents are required during sample processing, pneumatic pressure is used to burst the top and bottom foils. The pressure applied to the reservoirs first causes the top foils to rupture. Next, the pressure causes the bottom foils to rupture, releasing the contents of the reservoirs.

Lyophilized reagents include PCR mix, internal lane standard (ILS), and allelic ladder (which also contains ILS) and are preloaded in chambers within the integrated biochip. The PCR mix contains all components required for amplification, including primers, polymerase, deoxynucleotide triphosphates, magnesium ions, and buffer. Purified DNA from the eluate-holding chamber of the smart cartridge is utilized to reconstitute the lyophilized PCR cake prior to thermal cycling. The ILS and allelic ladder cakes are reconstituted prior to electrophoresis by PCR product/formamide and by formamide, respectively. The ILS (ILS600, Promega, Madison, WI) cake contains 22 fragments, ranging in size from 60 – 600 bases.

### DNA purification

A chaotrope-silica purification method was adapted for microfluidic DNA purification. The approach is based on guanidinium-mediated binding of DNA to silica, with guanidine-based lysis, ethanol-based wash, and Tris-EDTA-based elution solutions prepared essentially as previously described [14]. All solutions are pneumatically driven across a 5 mm^2^ silica membrane.

### Rapid, multiplexed STR amplification

The system’s STR assay targets 16 loci in a 19.5 min multiplexed PCR, as previously described [15]. Briefly, each of the five microfluidic reactions is performed in 7 μl, and the process consists of a 20-second denaturation at 94°C followed by 31 cycles of 4 seconds at 94°C, 15 seconds at 56°C, and 7 seconds at 70°C, followed by a final extension of 90 seconds at 70°C. The STR primer sequences are those of the PowerPlex® 16 kit (Promega Corporation, Madison, WI); they are the same primers purchased in bulk, but their concentrations differ from those of the PowerPlex kit to enable rapid amplification. One primer for each of the D3S1358, TH01, D18S51, D21S11, and Penta E loci is labeled with fluorescein; one primer for each of the TPOX, D8S1179, vWA, FGA, and amelogenin loci is labeled with carboxy-tetramethylrhodamine; and one primer for each of the D5S818, CSF1PO, D7S820, D13S317, D16S539, and Penta D loci is labeled with 6-carboxy-4’, 5’-dichloro-2’, 7’-dimethoxy-fluorescein. The ILS fragments are labeled with carboxy-X-rhodamine.

### Separation and detection

**Figure 2:**
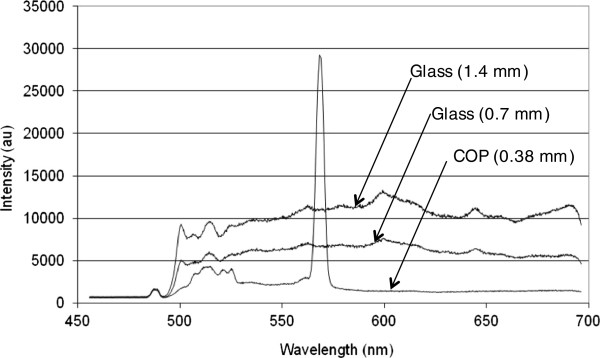
**Autofluorescence of cyclic olefin polymer and glass by excitation at 488 nm and measured across the visible wavelength spectrum between 470 and 700 nm.** The peak at about 570nm is the Raman emission peak of cyclic olefin polymer. The S&D biochip is fabricated from cyclic olefin polymer, allowing for low autofluorescence in a single-use component. By contrast, glass separation capillaries and biochips are typically reused, requiring more complicated instrumentation and leading to potential cross contamination.

The system separates and detects STR fragments in a process that consists of filling the separation channels with sieving matrix, filling the anode and cathode chambers with Tris-TAPS-EDTA electrophoresis buffer, performing pre-electrophoresis at 8 kV for 6 minutes, injection at 1.1 kV for 1.25 minutes, and separation at 4 kV for 30 minutes. The plastic S&D biochip component of the BCSC contains six independent microfluidic channels. Each separation channel has cross-sectional dimensions of 40 × 100 μm, and is 225 mm long. The sieving matrix is an aqueous solution of 4% (w/v) linear polyacrylamide; high molecular weight linear polyacrylamide was fabricated by polymerizing Acrylamide (GE Healthcare, Piscataway, NJ) in the presence of N,N,N’,N’-tetramethylethylenediamine (Sigma, St. Louis, MO) and ammonium persulfate (Sigma). When detecting fluorescently labeled fragments in plastic substrates, it is important to minimize the autofluorescence characteristics of the plastic. The S&D biochip is fabricated from cyclic olefin polymer with a thickness of 376 μm. Figure 2 shows that autofluorescence of this polymer is much lower than that of glass.

### Signal processing and expert system software

Signal processing is initiated automatically at the conclusion of S&D. Processing consists of a series of functions including raw data capture, peak identification, and placement of fragments into separate dye colors. The baseline subtraction algorithm applies a sliding window across the raw electropherogram and at each point determines the minimum signal strength within the window. The width of the sliding window was selected to be five alleles wide. Applying this algorithm to the raw data results in the generation of the signal baseline. This baseline is subtracted from the raw data to generate a baseline-subtracted electropherogram. Spectral separation is performed by: (1) applying a peak-finding algorithm to identify peaks on the baseline-subtracted electropherogram; (2) determining the ratio of the signal strengths of the four detectors for each peak; (3) grouping the peaks by their detector (color) ratios into a four-color ratio matrix; and (4) applying a color correction matrix (the inverse of the color ratio matrix) to the baseline-subtracted electropherogram to generate a spectrally separated electropherogram. The resulting electropherogram displays the signals from the photomultiplier detectors of the instrument. Each photomultiplier is a discrete detector with an independently set gain. The gains are not scaled, and, accordingly, the scales for each color are slightly different.

The expert system is an automated allele calling software that interprets the processed data based on a set of rules designed to reproduce the analytical processes of a forensic analyst without requiring human intervention. The product of the expert system is an electropherogram presented as a bitmap file (.bmp). The DNA peaks in the electropherogram are labeled with allele calls in gray boxes if the results clearly meet the calling rules and in red boxes if the results are questionable and require an analyst’s review.

The expert system analyzes the baseline-subtracted, spectrally separated electropherogram. Expert system parameters were determined empirically after reviewing results from several thousand samples processed in the fully automated system. Settings used in profile determination were selected to minimize the number of erroneous allele designations rather than to consider only maximization of the number of correct calls. This approach results in some ‘no-call’ loci (that is, those that do not generate confident allele designations) and resulting partial profiles. The allele candidate designations for these loci are labeled in red boxes in the .bmp electropherogram output display but are not reported to the .cmf file.

Fragments are not considered or reviewed unless they are >150 rfu above the center of the noise. Some fragments are immediately rejected from consideration for allele designation, for example, spikes (constituting no more than two raw collection scans) or dye blobs (more than twice the width of a nominal fragment). Comparison of amplified sample fragment migration with migration of a set of 20 fragments (ILS) of known size of 80 to 550 bases is used to designate the size of each sample fragment. These values are compared with sizes of known components of an allelic ladder to translate sample fragment size into ‘candidate allele designations’, using rules defined by the International Society of Human Genetics (formerly the International Society for Forensic Haemogenetics) [16,17]. If the allelic ladder fails in a given run, a fixed set of sizing bins is used for designation.

The software then quarantines some fragments as suspect or rejected based on several criteria including (1) stutter fragment, (2) incomplete nucleotide template addition (iNTA) fragment, (3) insufficient peak height, (4) insufficient peak height ratio in a heterozygous locus, and (5) excessive peak height ratio (explained below) in a homozygous locus. Incomplete nontemplated addition is considered initially. Any fragment that is less than 20% the height of a fragment approximately one base larger is considered an iNTA fragment and ‘not an allele’. Stutter candidates are limited to those that are one repeat shorter than another allele candidate (called the parent allele candidate) and are known not to be the result of a spike, pull up, or iNTA. The locus-specific rules of allele designation, described next, are used to exclude the peak-height-adjusted stutter peak candidate alleles from allele designation.

The two remaining allele candidates with the highest peak heights within one locus, after removal of allele candidates with the rules already listed, are tested first for heterozygote peak height ratio status, and then for homozygote peak height ratio status. If together the alleles fail both tests, the output is considered inconclusive (that is, a ‘no call’), the .bmp labels of all alleles for the locus are shown in red boxes rather than the typical gray boxes, and the locus profile is not reported to a .cmf file. Heterozygote loci contain two allele candidates that (1) both exceed peak heights of 250 rfu and (2) have a peak-height ratio greater than 0.37 (that is, the peak height of the lower peak divided by the peak height of the higher peak exceeds 0.37). Homozygote loci contain either one allele candidate or two allele candidates that (1) failed the heterozygote test, (2) have a higher peak exceeding 600 rfu, and (3) have a peak-height ratio less than 0.20 (that is, the peak height of the lower peak divided by the peak height of the higher peak is less than 0.20). Note that if there is no second peak in the homozygote test, the last value is 0.00 for this determination.

No user action is required to create or analyze the output files. The software outputs several files, including a .bmp file displaying the electropherogram, an .fsa file to permit evaluation of the output in other software programs, and a .cmf file to permit direct data upload to CODIS-compatible databases by an authorized user. Partial profiles are exported as .bmp and .fsa files, but only partial profiles with at least ten called CODIS loci are exported as .cmf files. Finally, note that for buccal swab analyses (presumed to be from a sole source), the software also rejects entire samples displaying evidence of a mixed sample, such as two or more loci each containing three or more alleles.

### Instrument overview

**Figure 3:**
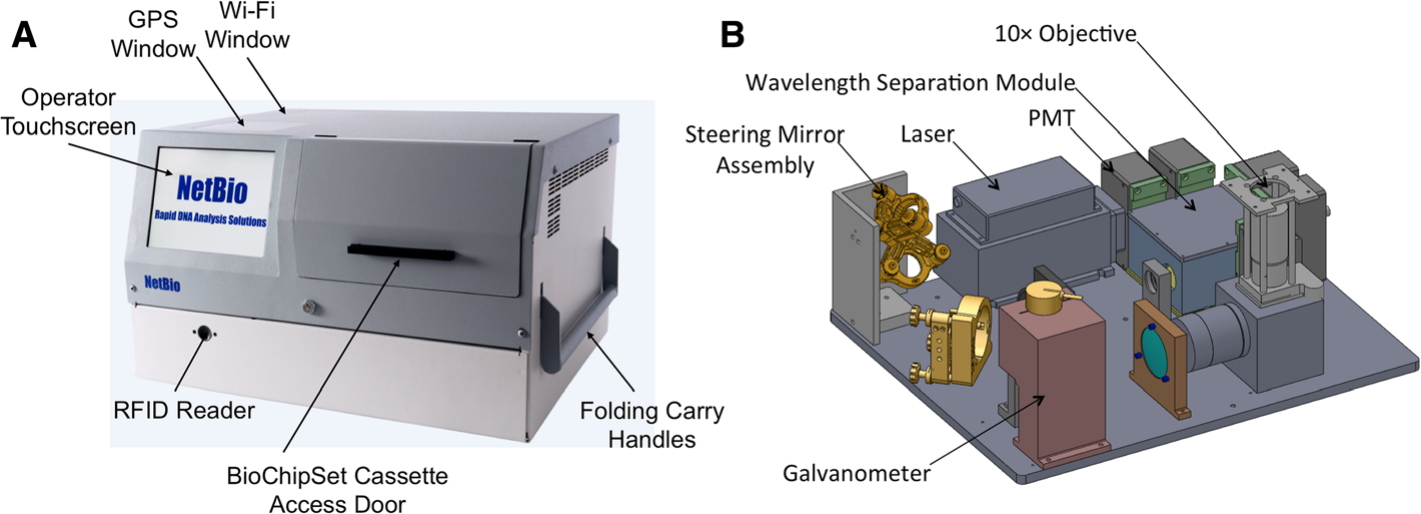
**The fully integrated instrument. (A)** The instrument weighs 50 kg (two-person lift), has dimensions of 26.6” × 16.5” × 23.1”, and is ruggedized to MIL-STD 810F for transportation vibration and shock. **(B)** Rendering of the optical train inside the instrument. For excitation, laser light is directed to the galvanometer via the steering mirror assembly. The galvanometer interrogates each of the six separation channels of the S&D biochip, exciting fluorophores bound to the amplified STR fragments. For detection, fluorescence is collected by the objective and directed to the photomultiplier tubes.

The fully integrated instrument (Figure 3A) is ruggedized to MIL-STD 810F for transportation vibration and shock, weighs 50 kg (portable by a two-person lift), and has dimensions of 26.6” × 16.5” × 23.1” (676 × 419 × 587 mm). It can operate with a supply voltage of between 90 and 260 V AC at 50 or 60 Hz, draws 4.5 amps (120 V AC at 60 Hz) at peak load, and can run on standard military field generators. The instrument comprises a set of subsystems, including an optical subsystem for exciting and detecting fluorescently labeled STR fragments during electrophoresis, a high-voltage subsystem for electrophoresis, a thermal subsystem [15] for multiplexed amplification, a pneumatic subsystem to drive fluids throughout the BCSC, and a ruggedization subsystem to allow transport and field-forward operation without recalibration or optical realignment. A single-board computer that is integrated with the instrument controls subsystem functions, performs data processing, interfaces with the user through an integrated touchscreen, and provides ethernet, wireless 802.11, and USB connectivity. An integrated global positioning system provides position and time data.

### Optical subsystem

The optical subsystem (Figure 3B) generates results by exciting the dye molecules of the labeled STR fragments and then detecting the emitted fluorescence. Excitation is achieved with a laser that emits at a wavelength of 488 nm and generates 200 mW of output power (Coherent, Santa Clara, CA). Emitted fluorescence is separated by wavelength by a set of dichroic mirrors and bandpass filters (Omega Optical, Brattleboro, VT), and detected by a set of four photomultipliers (Hamamatsu, Bridgewater, NJ). The instrument also accepts a fifth photomultiplier for alternative assays. Laser excitation is transmitted to the S&D biochip detection window and emitted fluorescence is transmitted to the photomultiplier tube detectors by a free-space optical system consisting of a set of lenses, mirrors and a custom 10× objective. A galvanometer (Cambridge Technologies, Lexington, MA) positioned in the beam path directs the laser excitation, and collects fluorescence from each of these channels individually. A lane-finding algorithm is applied to align the optical system automatically to each of the channels within the S&D biochip. Excitation and detection is performed at 5 Hz.

### High-voltage subsystem

A high-voltage subsystem applies up to 10 kV (Spellman, Hauppauge, NY) to the separation channel of the S&D biochip to generate an electric field that moves the STR fragments along the separation channel. This voltage is applied to the S&D biochip through a set of spring-loaded electrodes integrated on the biochip holder of the instrument. In addition, these spring-loaded electrodes are coupled to the anode and cathode electrodes on the BCSC when it is inserted into the instrument.

### Pneumatic subsystem

The pneumatic subsystem is the primary drive mechanism used by the instrument and is responsible for actuating reagent release and transporting fluids from one portion of the BCSC to another. The avoidance of mechanical, magnetic, centrifugal, or other drive mechanisms reduces the number of moving parts in the system to enhance ruggedization and robust operation in field-forward settings. When a run is initiated, the air compressor fills a set of pressure tanks (Bimba, University Park, IL) to 100 psi (0.689 MPa). The compressed air is routed through an electronically controlled pressure regulator, a set of solenoid valves (Humphreys, Kalamazoo, MI), and pneumatic tubing to a pneumatic manifold that is mounted on the instrument. The instrument manifold and the pneumatic ports of the BCSC are coupled when the BCSC is inserted into the instrument. The pneumatic system allows each of the pneumatic and fluidic lines within the BCSC to be activated and driven at a programmed pressure. Pneumatic pressures of 50 psi (0.344 MPa) are applied to actuate reagent release. Pneumatic pressures of between 1 to 5 psi (0.007 to 0.0344 MPa) are applied for fluidic transport within the BCSC. A high-pressure system is utilized to load sieving matrix through the separation channels at approximately 300 psi (2.07 MPa).

### Ruggedization subsystem

The components of the instrument that are most sensitive to shock and vibration are those of the optical subsystem. Accordingly, all optical elements are mounted to a base plate and isolated from shock and vibration through a set of mounts. The instrument has an automated lane-finding capability that automatically aligns the optical system to the separation channels of the S&D biochip prior to STR fragment detection, to compensate further for any movement within the optical train during transport. Lane finding is performed by scanning laser light across the separation channels within the detection window to generate a waveform of reflected intensity with scanner position. The location within the waveform characteristic of the centers of each separation channel is identified and applied. The ruggedization components and automated lane-finding system allow the instrument to be transported without the requirement for a manufacturer’s recalibration or optical realignment.

### System operation and sample tracking

Sample collection is performed using a DNA-free cotton-tipped swab held in place by a locking plastic cap. The cap contains a RFID chip for sample tracking within the instrument. To initiate a run, the operator logs onto the instrument using the touchscreen. The touchscreen provides visual prompts to place the RFID-labeled cap of a sample in front of the RFID scanner of the instrument, to insert the swab into the BCSC and to enter a sample ID. Once a swab is placed into the BCSC, it is securely and irreversibly locked in place. The locking mechanism ensures that a sample is not removed following placement to avoid cross contamination and to maintain a closed system. Following the loading of the fifth swab, the instrument door opens, and the touchscreen prompts the user to insert the BCSC into the instrument and close the door to initiate sample processing. Within the instrument, an internal RFID scanner reads the five RFID-labeled caps and identifies the lane position in which each sample was placed, linking the sample to its lane position and to the profile that will be generated by that lane, completing the traceable connection between sample identification and placement and the STR profile. There is no need to place the swabs in any particular order within the BCSC.

Nontechnical employees performed 15% of the fully integrated runs reported here. Less than 30 minutes of training was provided: the nontechnical operators switched on the instrument, logged on, inserted swab samples into BCSCs, loaded BCSCs into the instrument, initiated runs (by closing the BCSC door), and removed the BCSC following run completion. All runs performed by nontechnical operators were completed successfully, with no difference in results noted between runs performed by technical and nontechnical operators.

Conventional samples for concordance testing were processed using the PowerPlex 16 HS system and analyzed by Cellmark Forensics, LabCorp Specialty Testing Group (Dallas, TX).

## Results and discussion

### Process overview

**Figure 4:**
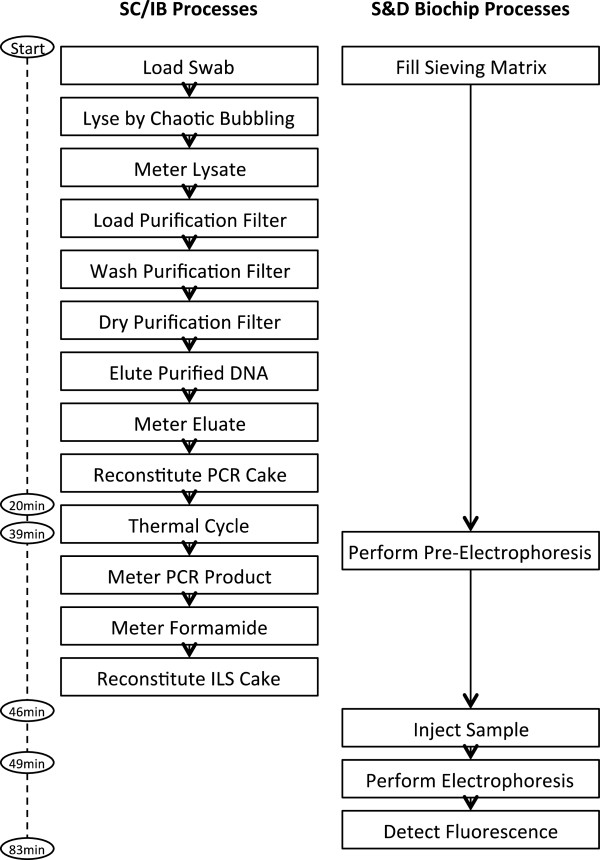
**Flowchart of process steps during a system run.** The entire process from inserting samples to displaying called profiles is 84 minutes. Two sets of processes occur in parallel: preparation of the sample for electrophoresis and preparation of the separation channels for electrophoresis.

Figure 4 shows a flowchart of the processes that occur during an instrument run. Following insertion of the BCSC into the instrument, bursting of reagent foils takes place. At this point, two parallel processing scripts are initiated, one to process the samples and the other to prepare the S&D biochip for electrophoresis. For sample preparation, cells are first subjected to a guanidine-based bind-wash-elute protocol. Following elution, the purified DNA is metered and utilized to reconstitute the PCR cake, and rapid thermal cycling is conducted. Following thermal cycling, amplified product is joined with formamide and ILS, and each sample is now ready for electrophoresis. In parallel during sample processing, sieving matrix is transferred from the gel smart cartridge to the separation channels of the S&D biochip followed by pre-electrophoresis. Each sample for electrophoresis is transferred to a cathode chamber, and electrophoresis is conducted with labeled STR fragments ultimately detected at the S&D window. Signal processing and profile generation are then performed, using automated expert-system software. The entire process from inserting samples to displaying called profiles is 84 minutes.

### DNA purification and amplification

**Figure 5:**
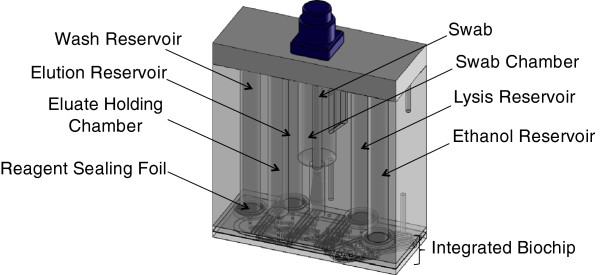
**Single unit of the smart cartridge.** Each unit contains four reagent reservoirs (for lysis, wash, ethanol, and elution solutions), with each reservoir sealed by top and bottom foils. Fluids move from chamber to chamber via channels in the integrated biochip. For example, lysis buffer and ethanol travel via the integrated biochip to the swab chamber, where chaotic bubbling and cell lysis occur. At the conclusion of the purification process, DNA is transferred from the eluate-holding chamber to the integrated biochip, where it reconstitutes a lyophilized PCR cake.

Figure 5 shows a single unit of the smart cartridge. Following swab introduction into the swab chamber, lysis solution is driven through a small hole in the swab chamber at high pressure, generating turbulent flow. The turbulence of this chaotic bubbling around the swab creates shearing forces on the cells, promoting rapid and efficient cell lysis within a few seconds and without heating.

From the swab chamber, the lysate is passed to a holding chamber in the integrated biochip, where approximately 25% of the total lysate is passed through the purification filter to capture the DNA. Then the captured DNA is washed to remove impurities, and the purified DNA is eluted and transferred to a metering chamber in preparation for amplification. In developing the purification module, two issues were considered. First, the quantity of DNA present on a typical buccal swab may be of the order of hundreds to thousands of nanograms, two or three orders of magnitude in excess of that required for amplification. This disparity was addressed by a number of features, particularly (1) the use of only 25% of the lysate, as noted, and (2) diluting the DNA during the elution process. The second issue is that the quantity of DNA present on a buccal swab is highly variable. To overcome a need for quantification, the purification process makes use of a silica filter that has an effective volume so small that it has only a low capacity to bind DNA (approximately 200 ng), serving to compress the range of DNA bound and eluted. Using the guanidine method on swabs in tube-based experiments designed to maximize DNA recovery, buccal swabs were found to contain 1266.8 ng DNA (713.7 standard deviation, *n* = 90), with an approximately 15-fold range (304.8 to 4455.3 ng/swab). Using the features discussed in the BCSC, the total amount of DNA eluted is reduced by almost a factor of ten to a mean of 133.2 ng (45.4 standard deviation, *n* = 145). Furthermore, the range is narrowed to approximately 4-fold (67.8 to 234.1 ng/sample). The eluted DNA is used to reconstitute the lyophilized PCR cakes, and rapid microfluidic PCR is performed in 7 μl reaction chambers, as described. Finally, purifying the DNA using the microfluidic chaotrope-silica method allows the generation of full STR profiles.

### Reagent stability

**Figure 6:**
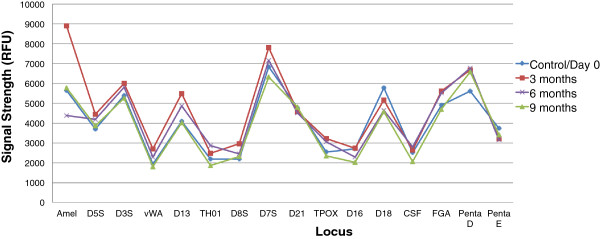
**Stability of lyophilized PCR cakes stored at 30°C.** The *y*-axis is signal strength (rfu/allele taken from average of *n* = 8 replicates per time point); the *x*-axis displays the 16 allelic loci from the Powerplex16 multiplex panel. The signal strength of 9-month stored cakes is comparable with that of the control.

All reagents have been shown to be stable for at least 6 months at 22°C in tubes made of identical materials to the BCSC, and experiments to assess longer-term stability in BCSCs are ongoing. Liquid PCR mix is known to be quite unstable, and, accordingly, was the initial focus of lyophilization efforts. Stability studies using the lyophilized PCR cakes were performed by incubating the cakes in a 30°C oven. Their stability was evaluated by performing rapid microfluidic amplification using standard 9947A genomic DNA (MCLAB, South San Francisco, CA) as a template. Figure 6 is a plot of the signal strength of alleles across 16 loci (Powerplex®16 System, Promega, Madison, WI) and shows that the PCR cakes are stable for at least 9 months. Studies to demonstrate stability beyond this time frame are ongoing.

**Figure 7:**
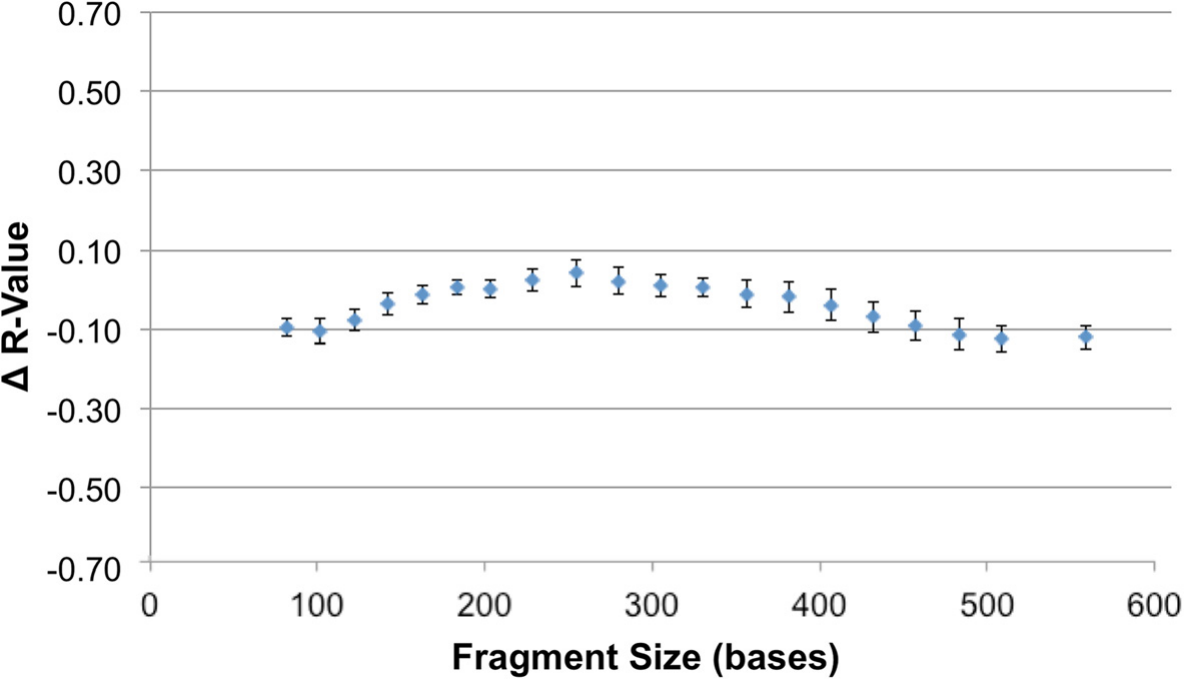
**Stability of S&D reagents stored at 22°C.** Six batches each of sieving gel matrix and electrophoresis buffer were stored for six months. *R* was calculated based on the peak separation and their base widths. The changes in *R* over the six months are plotted (± standard deviation), demonstrating that the reagents are stable for at least six months.

The performance of purification reagents was measured by the quantity and purity of DNA and functionally by microfluidic amplification. Following purification reagent storage in environmental control chambers at 22°C, DNA was purified from buccal swabs from several donors (*n* = 12) using foil-sealed reagents in cyclic olefin polymer and compared with control glass bottle cap-sealed reagents. Mean DNA yield obtained from fresh reagents (day 0), 6-month-old control reagents (stored in bottles at 22°C), and 6-month-old foil-sealed reagents were 1216 ± 540 ng, 969 ± 380 ng, and 1120 ± 520 ng, respectively. Highly pure nucleic acid was obtained in all sets with *A*_260_/*A*_280_ of 1.95 ± 0.07 for the 6-month-old foil-sealed reagents. Purified DNA solutions from all donors were diluted to 0.4 ng/μl and used to resuspend lyophilized PCR cakes for microfluidic amplification (approximately 2 ng DNA per 7 μl amplification reaction). The signal strength of alleles across all 16 loci from the 6-month stability data set showed comparable efficiency (peak heights within 20%) between the control and the foil-sealed reagent sets (at both day 0 and 6 months) suggesting that neither DNA degradation nor PCR inhibition occurred during storage. Finally, S&D reagent stability was performance tested based on size resolution and signal strength of the ILS marker. Sieving gel matrix and electrophoresis buffer were placed in cyclic olefin polymer tubes, sealed with foil, and placed in an environmental control chamber at 22°C. Resolution (Figure 7) and signal strength were stable for at least 6 months.

### Sample-in to results-out performance of the fully integrated system

**Figure 8:**
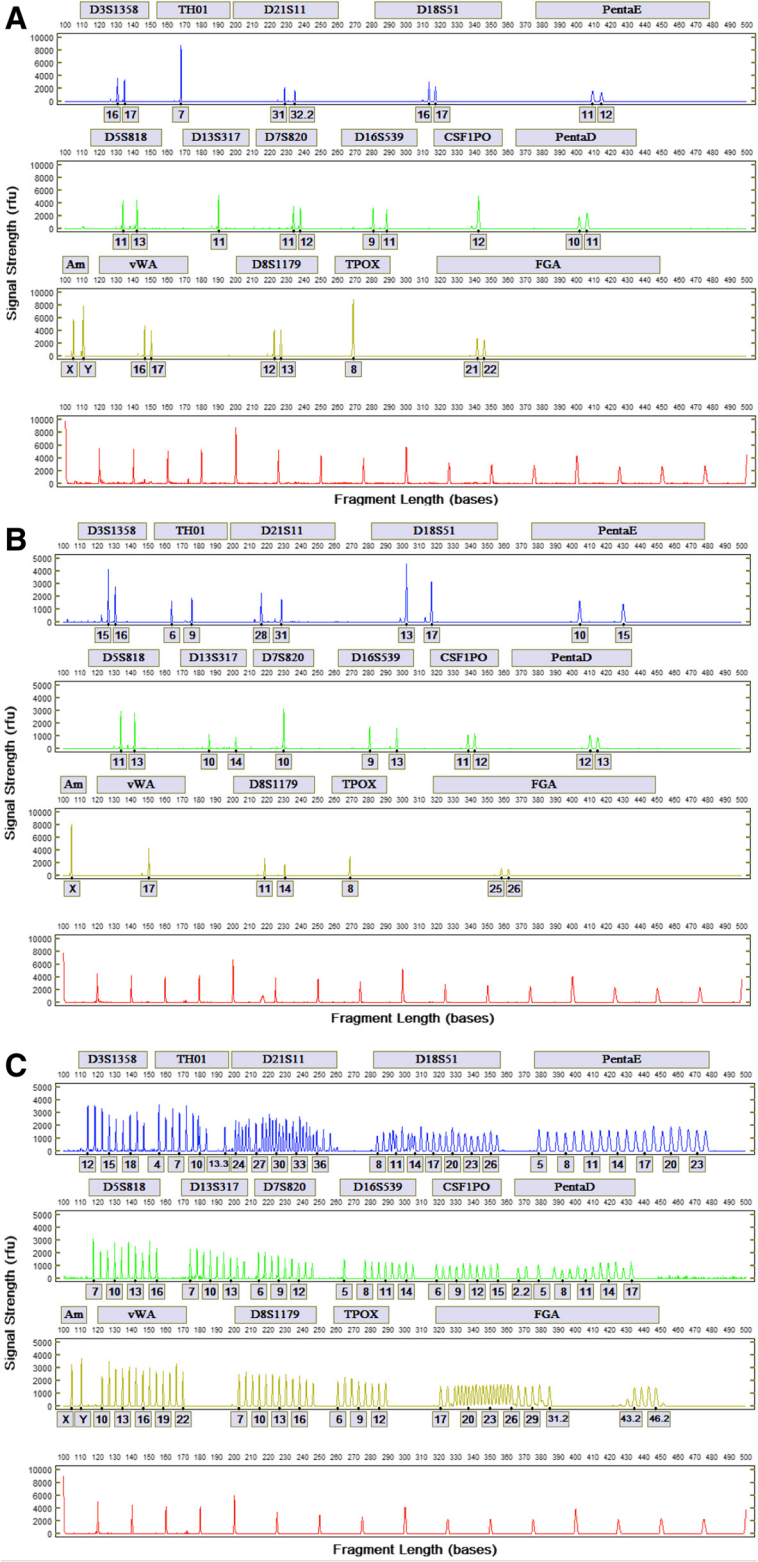
**Electropherograms generated from a fully integrated run.** Full PCR/STR profiles generated from buccal swabs of male **(A)** and female **(B)** donors; and control allelic ladder **(C)**.

Figure 8 shows electropherograms of three samples from a fully integrated run generated from a buccal swab of a male donor, a buccal swab of a female donor, and the allelic ladder. The expert system called full profiles from both donors, and the profiles are concordant with Powerplex16 profiles generated using conventional procedures. A set of 100 buccal samples was processed on the fully integrated system and the .cmf files generated were compared with those from samples processed and analyzed conventionally by an outside laboratory. Of these samples, 85 generated full CODIS profiles, five generated partial CODIS profiles (four with 12 loci and one with 11 loci), and ten generated no profiles. In one sample, a spike was designated as an allele. With this exception, the results demonstrated 100% concordance with those from the conventionally processed samples (and greater than 99.95% allelic concordance when the spike was included). The expert system has since been modified to prevent calling of electrical spikes, rare events characteristic of high-voltage electrophoresis systems. The five partial profiles had random match probabilities ranging from one in 11 trillion to one in 364 quadrillion, demonstrating that these profiles are also useful for searching.

**Figure 9:**
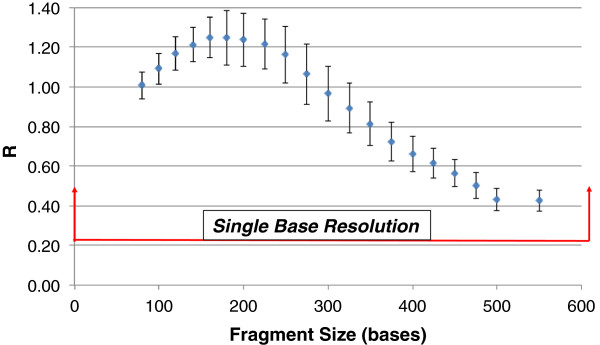
**Resolution of the system.** Resolution (± standard deviation) was determined for 119 samples. *R* was calculated based on the peak separation and their base widths as described [18]. *R* of 0.3 or greater represent single-base resolution at a given fragment size, and single-base resolution is achieved from 100 to greater than 500 bases.

**Figure 10:**
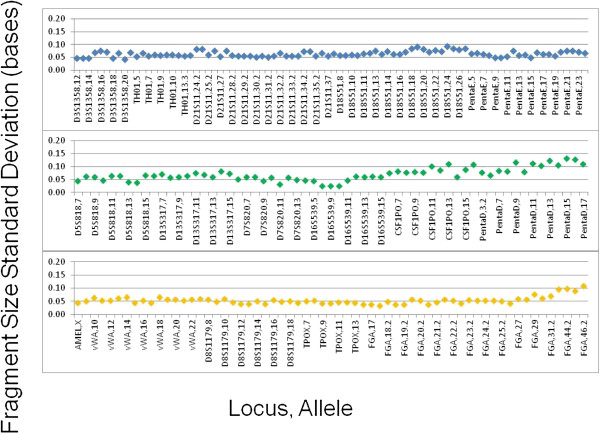
**Inter-run precision of the fully integrated system was determined based on 20 allelic ladder runs.** Standard deviations and alleles are presented for each of three fluorescent dyes. The observed precision is expected to be sufficient for calling of off-ladder alleles.

**Figure 11:**
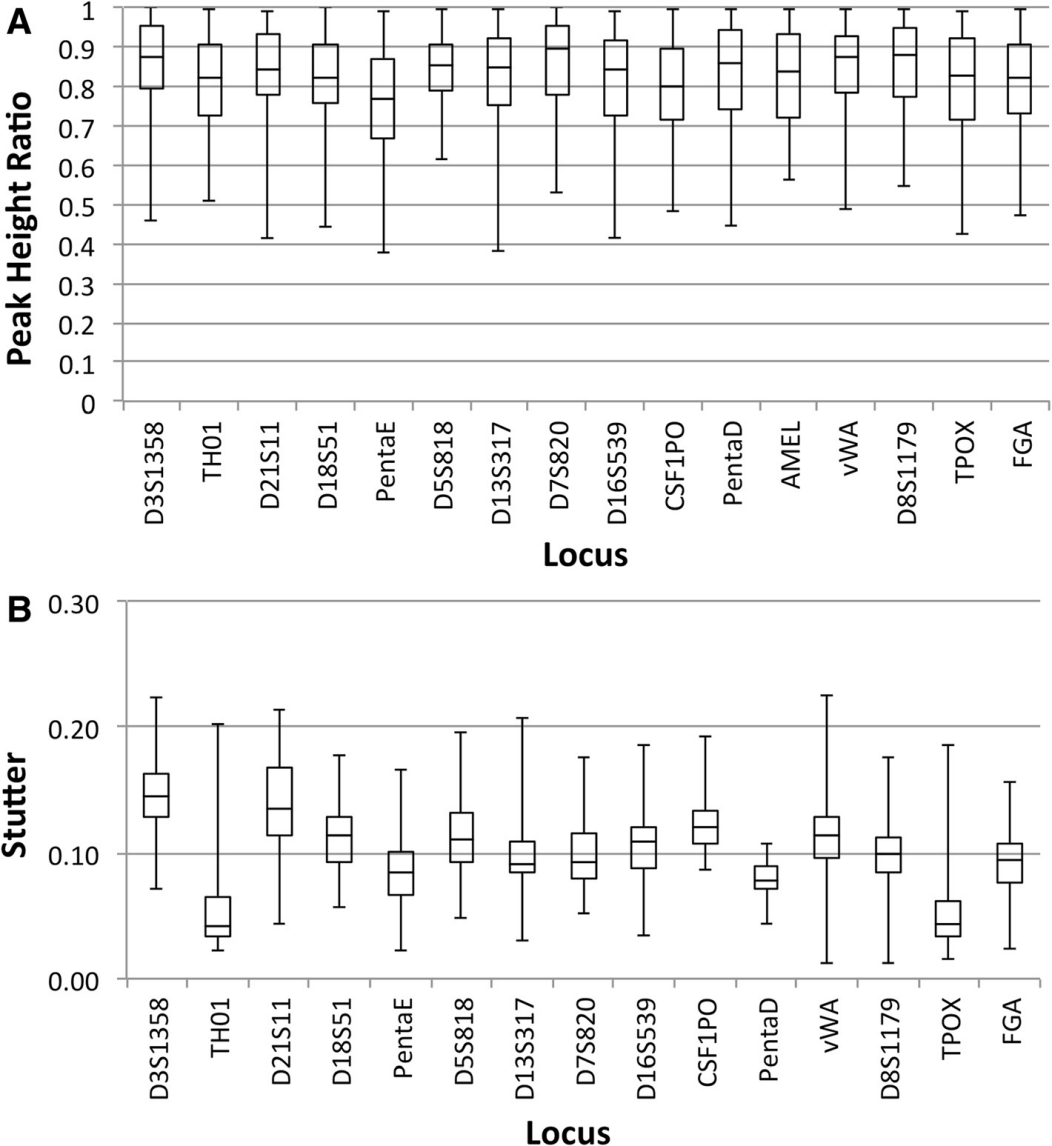
**Peak-height ratios (A) and stutter (B).** Determined from 90 buccal swab samples processed on the fully integrated system.

The resolution of the rapid DNA analysis system is presented in Figure 9 based on calculation of *R* for 119 samples. The system exhibits single-base resolution across the entire range of separation, from 100 to greater than 500 bases. Figure 10 shows inter-run precision based on 20 allelic ladders generated by the system. Precision is characterized by a standard deviation of ±0.05 - 0.10 bases for most alleles, with standard deviation approaching ±0.15 bases for some of the Penta D alleles. This degree of precision demonstrates the sizing reproducibility of the system. Figure 11A and 11B show peak-height ratios and stutter, respectively, from 90 buccal samples. The iNTA measurements (not shown) are almost always less than 5% of the major peak, with less than 1% of the measurements showing iNTA of 5 to 10% of the major peak.

### System ruggedization testing

To perform MIL-STD shock testing, instruments were subjected to a transport drop test. Crated instruments were raised above a solid concrete floor with a hoist and dropped 30 cm. A bench drop test was performed by placing uncrated instruments on a laboratory benchtop and raising one side with a 10 cm spacer. The spacer was removed quickly causing the side of the instrument to fall, and this test was performed on each of the four sides of the instruments. Prior to and following each type of test, a series of automated evaluations was performed on the thermal cycler, laser, galvanometer, high-voltage power supply, photomultiplier tubes, and pneumatics subsystems. Pre- and post-testing telemetry showed that each of the subsystems was functional and performed as designed following shock. For vibration testing, the instruments were restrained on a vibration table and subjected to a low-frequency vibration sweep. For shock and vibration testing, post-test runs of swab samples demonstrated that the system generated STR profiles as designed.

## Conclusions

The system described here allows fully automated, fully integrated processing of buccal swab samples in 84 minutes. The system has several features that may prove valuable in both field-forward and laboratory settings. In particular, the use of a single consumable minimizes operator requirements and allows a nontechnical user to perform system runs. Ease of use for a nontechnical operator is of particular importance outside the laboratory, whether at a police-station booking desk, on the battlefield, or at a border or port. Similarly, long-term stability, at and above room temperature, of the reagents used in the BCSC and ruggedization to MIL-STD 810 are critical for out-of-laboratory testing. The system currently generates full STR profiles for 85% of input samples. Sample failures are typically caused by blockages in channels that prevent amplification or electrophoresis and, accordingly, prevent generation of an STR profile. Work to enhance manufacturing quality control processes to such fluidic failures is in progress.

It is expected that as rapid DNA analysis matures, the approach will lead to enhanced technical capabilities. For example, the addition of a quantification module might be critical for the analysis of casework samples. Furthermore, the PCR module of the system described here can be used for DNA sequencing and has the potential to be applied to mitochondrial DNA analysis. Similarly, we have developed a 27-locus multiplex PCR assay (based on detection using a six-color optical system modified from that presented here) that simultaneously interrogates 26 STR loci plus the amelogenin locus in human genomic DNA samples [19]. These include the United States CODIS 13 core STR loci, the European standard 15 STR loci plus amelogenin overlapping with 7 STR loci in the core CODIS set, STR loci of a recently proposed CODIS expanded standard core set that contains 20 required and three recommended STR loci [20,21], the D6S1043 locus commonly used in China [22], and the Penta C, Penta D, and Penta E loci [23]. In addition to expanded assay capability, the BCSC and instrumentation can be enhanced in many ways, including miniaturization, faster processing time, incorporation of single nucleotide polymorphism and sequencing analyses, and integration with other biometric modalities.

Several steps will be required before STR profiles generated by rapid DNA analysis may be utilized to search the National DNA Index System. From a technical standpoint, developmental validation will be essential; the fully integrated system includes the BCSC, instrumentation, and expert system software, all of which will be validated and tested for concordance against conventional modular technologies. Just as importantly, certain policies, and, in some jurisdictions, laws will need to be modified to allow rapid DNA analysis to be utilized in law-enforcement settings outside the laboratory. Toward this end, the FBI’s Scientific Working Group on DNA Analysis Methods has established a Rapid DNA Committee to evaluate and establish principles of validation, and recommend revisions to the FBI’s Quality Assurance Standards for rapid DNA analysis [24].

In the recently decided Supreme Court case Maryland v King, the taking and analyzing of a buccal swab was held to be ‘a legitimate police booking procedure that is reasonable under the Fourth Amendment’ [25]. The STR profile generated at the booking desk could be utilized to query a database and lead to a determination of whether or not to release the arrestee. Access to search the National DNA Index System is currently limited to laboratories that comply with the FBI’s Quality Assurance Standards and the DNA Identification Act of 1994, and searching can only be performed at designated times. Accordingly, critical steps toward the implementation of rapid DNA analysis outside the forensic laboratory concern review and revision of laws and policies related to the sites that may access Federal and State DNA databases, the timing of and conditions under which access may be granted, security of STR data generated outside the laboratory, and mechanisms to ensure privacy of the individuals involved (many of whom will be determined not to have committed a crime).

## Abbreviations

ANDE: Accelerated nuclear DNA equipment; BCSC: BioChipSet cassette; ILS: Internal lane standard; iNTA: Incomplete nucleotide addition; PCR: Polymerase chain reaction; RFID: Radio-frequency identification; rfu: Relative fluorescence unit; S&D: Separation and detection; STR: Short tandem repeat.

## Competing interests

All authors are employees of and shareholders in NetBio.

## Authors’ contributions

RST, CH, SV, and LP carried out the molecular biology studies. JWS developed the multiplexed STR assay and the analytic software, and ET and RFS conceived the overall approach and directed the study. ET, RST, JWS, and RFS wrote the manuscript, and all authors read and approved of the final manuscript.

## References

[B1] Jeffreys AJ Wilson V Thein SL Hypervariable ‘minisatellite’ regions in human DNA Nature 1985 314 67 73 10.1038/314067a0 3856104

[B2] Jeffreys AJ Wilson V Thein SL Individual-specific ‘fingerprints’ of human DNA Nature 1985 316 76 79 10.1038/316076a0 2989708

[B3] Jeffreys AJ Brookfield JFY Semeonoff R Positive identification of an immigration test-case using human DNA fingerprints Nature 1985 317 818 819 10.1038/317818a0 4058586

[B4] Weber JL May PE Abundant class of human DNA polymorphisms which can be typed using the polymerase chain reaction Am J Hum Genet 1989 44 388 2916582 PMC1715443

[B5] Litt M Luty JA A hypervariable microsatellite revealed by *in vitro* amplification of a dinucleotide repeat within the cardiac muscle actin gene Am J Hum Genet 1989 44 397 2563634 PMC1715430

[B6] Rassmann K Schlotterer C Tautz D Isolation of simple-sequence loci for use in polymerase chain reaction-based DNA fingerprinting Electrophoresis 1991 12 113 118 10.1002/elps.1150120205 2040259

[B7] Edwards A Civitello A Hammond HA Caskey CT DNA typing and genetic mapping with trimeric and tetrameric tandem repeats Am J Hum Genet 1991 49 746 756 1897522 PMC1683171

[B8] Budowle B **Studies for selecting core STR loci for CODIS.** In Proceedings of Cambridge Healthtech Institute’s Second Annual Conference on DNA Forensics: Science, Evidence and Future Prospects McLean, VA:: Cambridge Health Tech Institute; 1997

[B9] Moretti T Budowle B The CODIS STR project: evaluation of fluorescent multiplex STR systems Proceedings of the 50th Annual Meeting of the American Academy of Forensic Sciences 1998 San Francisco, CA: American Academy of Forensic Sciences

[B10] U.S. Department of Defense Biometric and Forensic Technology Forum[http://csis.org/event/us-department-defense-biometric-and-forensic-technology-forum]

[B11] Bienvenue JM Legendre LA Ferrance JP Landers JP An integrated microfluidic device for DNA purification and PCR amplification of STR fragments Forensic Sci Int Genet 2010 4 178 186 10.1016/j.fsigen.2009.02.010 20215029

[B12] El-Sissi O Franklin H Nielsen B Pagano S Belcinski R Bogdan G Jovanuvich S Apollo 200 fully integrated DNA HID system: multi channel results in under 2 hours J Biomol Tech 2011 22 S44

[B13] Hopwood AJ Hurth C Yang J Cai Z Moran N Lee-Edghill JG Nordquist A Lenigk R Estes MD Haley JP Integrated microfluidic system for rapid forensic DNA analysis: sample collection to DNA profile Anal Chem 2010 82 6991 6999 10.1021/ac101355r 20704389

[B14] Boom R Sol CJ Salimans MM Jansen CL Wertheim-van Dillen PM van der-Noordaa J Rapid and simple method for purification of nucleic acids J Clin Microbiol 1990 28 495 503 1691208 10.1128/jcm.28.3.495-503.1990 PMC269651

[B15] Giese H Lam R Selden R Tan E Fast multiplexed polymerase chain reaction for conventional and microfluidic short tandem repeat analysis J Forensic Sci 2009 54 1287 1296 10.1111/j.1556-4029.2009.01200.x 19840207

[B16] Bar W Brinkmann B Budowle B Carracedo A Gill P Lincoln P Mayr W Olaisen B DNA recommendations. Further report of the DNA Commission of the ISFH regarding the use of short tandem repeat systems. International Society for Forensic Haemogenetics Int J Legal Med 1997 110 175 176 10.1007/s004140050061 9274938

[B17] Lincoln P DNA recommendations: further report of the DNA Commission of the ISFH regarding the use of short tandem repeat systems Forensic Sci Int 1997 87 179 184 10.1016/S0379-0738(97)00110-2 9248037

[B18] Luckey JA Norris TB Smith LM Analysis of resolution in DNA sequencing by capillary gel electrophoresis J Phys Chem 1993 97 3067 3075 10.1021/j100114a038

[B19] Schumm J Gutierrez-Mateo C Tan E Selden RF A 27-locus STR assay to meet all United States and European law enforcement agency standards J Forensic Sci 2013 10.1111/1556-4029.12214 23822765

[B20] Hares DR Expanding the CODIS core loci in the United States Forensic Sci Int Genet 2012 6 e52 e54 10.1016/j.fsigen.2011.04.012 21543275

[B21] Hares DR Addendum to expanding the CODIS core loci in the United States Forensic Sci Int Genet 2012 6 e135 10.1016/j.fsigen.2012.01.003 21543275

[B22] Huang S Zhu Y Shen X Le X Yan H Genetic variation analysis of 15 autosomal STR loci of AmpFlSTR Sinofiler (TM) PCR amplification kit in Henan (central China) Han population Leg Med 2010 12 160 161 10.1016/j.legalmed.2010.02.004 20303817

[B23] Jacewicz R Jedrzejczyk M Ludwikowska M Berent J Polymorphism of pentanucleotide STR markers in Polish population sample Forensic Sci Int: Gen Suppl Series 2008 1 334 336 10.1016/j.fsigss.2007.10.055

[B24] Scientific Working Group on DNA Analysis Methods (SWGDAM): Validation Guidelines for DNA Analysis Methods [http://swgdam.org/SWGDAM_Validation_Guidelines_APPROVED_Dec_2012.pdf]

[B25] Supreme Court of the United States: Maryland v King 569 U.S. No. 12–207 (2013) [http://www.supremecourt.gov/opinions/12pdf/12-207_d18e.pdf]

